# Aspects of implementing a nurse-based follow-up of patients with liver cirrhosis: A qualitative study

**DOI:** 10.1371/journal.pone.0356782

**Published:** 2026-08-21

**Authors:** Maria Hjorth, Anna Forsberg

**Affiliations:** 1 Centre for Clinical Research in Dalarna, Uppsala University, Uppsala, Sweden; 2 Department of Public Health and Caring Sciences, Uppsala University, Uppsala, Sweden; 3 Department of Health, Medicine and Caring Sciences, Linköping University, Linköping, Sweden; 4 Institute of Health Sciences at Lund University, Lund, Sweden; 5 Department of Cardiothoracic Surgery, Skåne University Hospital, Lund, Sweden; University Hospital Cologne: Uniklinik Koln, GERMANY

## Abstract

**Aim:**

To explore aspects of implementing a nurse-based outpatient follow-up intervention for patients with liver cirrhosis by means of experiences reported by registered nurses, physicians and managers employed at six study clinics.

**Methods:**

This explorative, qualitative and deductive study involved interviews with 29 healthcare professionals and managers about the implementation of the intervention. Repeated interviews were performed from 2018 to 2022. Directed content analysis was performed, based on a coding scheme from the PARiHS conceptual framework. Reporting followed the COREQ guidelines.

**Results:**

The analysis revealed 23 different factors that influenced implementation of the nurse-based intervention, which covered all PARiHS elements. Eleven factors reduced the likelihood of successful implementation, e.g., the randomised study design became a moral dilemma, lack of clarity, resources and supportive leaders in the organisation, unclear roles, and a fragile mandate for nurses to implement the intervention. Twelve factors improved the probability for implementation success. The intervention was deemed safe, relevant and in line with clinical goals. Managers could facilitate the nurses’ role in the team, which supported the nurses’ mandate and professional growth.

**Conclusions:**

In conclusion, implementation of nurse-based interventions is facilitated by simple procedures that can be performed independently by RNs but also by the fact that the interventions are considered valuable for the clinic concerned. Staff shortage is a barrier to clinical research, and a moral dilemma might be experienced when not all patients in need of extensive care can be randomized to the intervention. An adequate number of benefits are needed to drive the success of an implementation. The PARiHS conceptual framework includes relevant perspectives required to implement evidence-based nursing practice.

## Introduction

Liver cirrhosis (LC) is a progressive chronic illness with high mortality rates due to serious complications, such as infections, variceal bleeding and hepatic encephalopathy [1]. Because of the increased occurrence of obesity, the disease is expected to become one of the top five non-communicable diseases in western countries [2]. Patients suffering from LC perceive that the current medically based care, as presented in Table 1, lacks caregiver continuity and a holistic perspective of their needs. Furthermore, it does not sufficiently support their self-management and learning process, which has consequences for patients’ daily life, self-management and prevention of complications [3]. In 2012, there was a global paradigm shift in LC management after new evidence revealed that the disease progression could be slowed down at an early stage [4]. This implied a change from treating complications on occurrence to introducing secondary prevention interventions, such as lifestyle modifications, which are one area of expertise among registered nurses (RNs) [5,6].

**Table 1:** Description of the standard of care routine and the changed follow-up routine implemented in the registered nurse-based outpatient Quality Liver Nursing Care Model intervention.

*Standardised medical follow-up for patients in the control group*	*Changed follow-up for patients in the intervention group*
Physician-based visits:	Standardised medical follow-up
Compensated disease^a^ 1–2 visits a year	Structured nurse-based visits:
Decompensated disease^b^2 to 4 visits a year	Compensated disease^a^ once yearly
Telephone consultation with RN^c^ or physician on demand	Ongoing decompensated disease^b^ 1–2 visits per month
Screening programme for hepatocellular cancer	Re-stabilised decompensated disease every 3 months
Screening programme for gastroesophageal varices	Content of the nurse-based visits:
Laparocentesis for ascites on demand	Person-centred communication
Inpatient care on acute occurrence	Assessment of current disease
	Information about self-care
	Self-care support

^a^Early LC stage with few symptoms.

^b^Advanced LC with ≥ 1 of the following symptoms: ascites, hepatic encephalopathy, gastroesophageal variceal bleeding.

^C^RN = Registered nurse.

Outpatient nursing interventions following LC have rarely been studied and implemented with uncertain results [7]. Previous interventions have had focus on symptom management in the advanced disease stage, with intention to reduce disease-related hospitalisations [8–10]. The 2012 year recommendations of secondary prevention following LC [4] motivated RN involvement directed to both patients in the stable asymptomatic disease stage as well as in the advanced disease stage. Accordingly, a RN-based LC outpatient intervention, called the Quality Liver Nursing Care Model (QLiNCaM) was developed, inspired by successful RN-based interventions for management of chronic heart failure [11], person centred care [12] and nursing theories (Table 1) [13]. The QLiNCaM was implemented and evaluated as part of a randomised controlled study at six Swedish hospitals from 2016 to 2022 (ClinicalTrials.gov NCT02957253) [14]. The intervention was delivered in a structured manner by trained RNs with positive effects on patient-reported and clinical outcomes, such as less inpatient care days, reduced mortality, improved quality of care and shorter time to identification of complications [15,16]. The primary outcome, health-related quality of life, was not affected [15]. In this paper, the intervention refers to the QLiNCaM, while the intervention study refers to the randomised controlled trial in which the RN-based QLiNCaM intervention was studied.

The intervention, which was characterised by an individualised follow-up routine and recommendations based on each patient’s unique needs, was deemed complex [14]. Furthermore, it required implementation in clinical practice at six hospitals with various clinical nursing research experiences and routines. Complex nursing interventions may be influenced by both facilitating and obstructing factors [17]. To understand how evidence of an intervention may be translated into clinical practice, determinants such as leadership, resources and knowledge require exploration. [18]. Results from this implementation study may consequently prevent failures and facilitate successful intervention of the QLiNCaM intervention at other hospitals in the future. Therefore, the intervention study was designed in line with the Medical Research Council guidelines for process evaluation [19], which include perspectives on implementation and influence of the context in which a change is implemented, which is the focus of the present study. The aim was to explore important aspects of implementing nurse-based outpatient follow-up of patients with LC by means of reports from RNs, physicians and managers employed at the six study hospitals. The purpose was led by the overarching research question: What aspects influenced implementation of the QLiNCaM intervention at the study clinics? Secondary research questions were as follows: What were the RN, physician and managers view of the evidence supporting the QLiNCaM intervention? What contextual factors did the RNs, physicians and managers experience as having influenced implementation of the QLiNCaM intervention? How did the contextual factors influence implementation of the QLiNCaM intervention? What factors did RNs, physicians and managers experience to have facilitated and or hindered implementation of the QLiNCaM intervention and the study performance?

## Materials and methods

### Design

This study employed an explorative design during the data collection phase. The data analysis was guided by directed content analysis [20], using the PARiHS conceptual framework [21] as a theoretical lens for the deductive-qualitative approach.

### Theoretical framework

**Fig 1:**
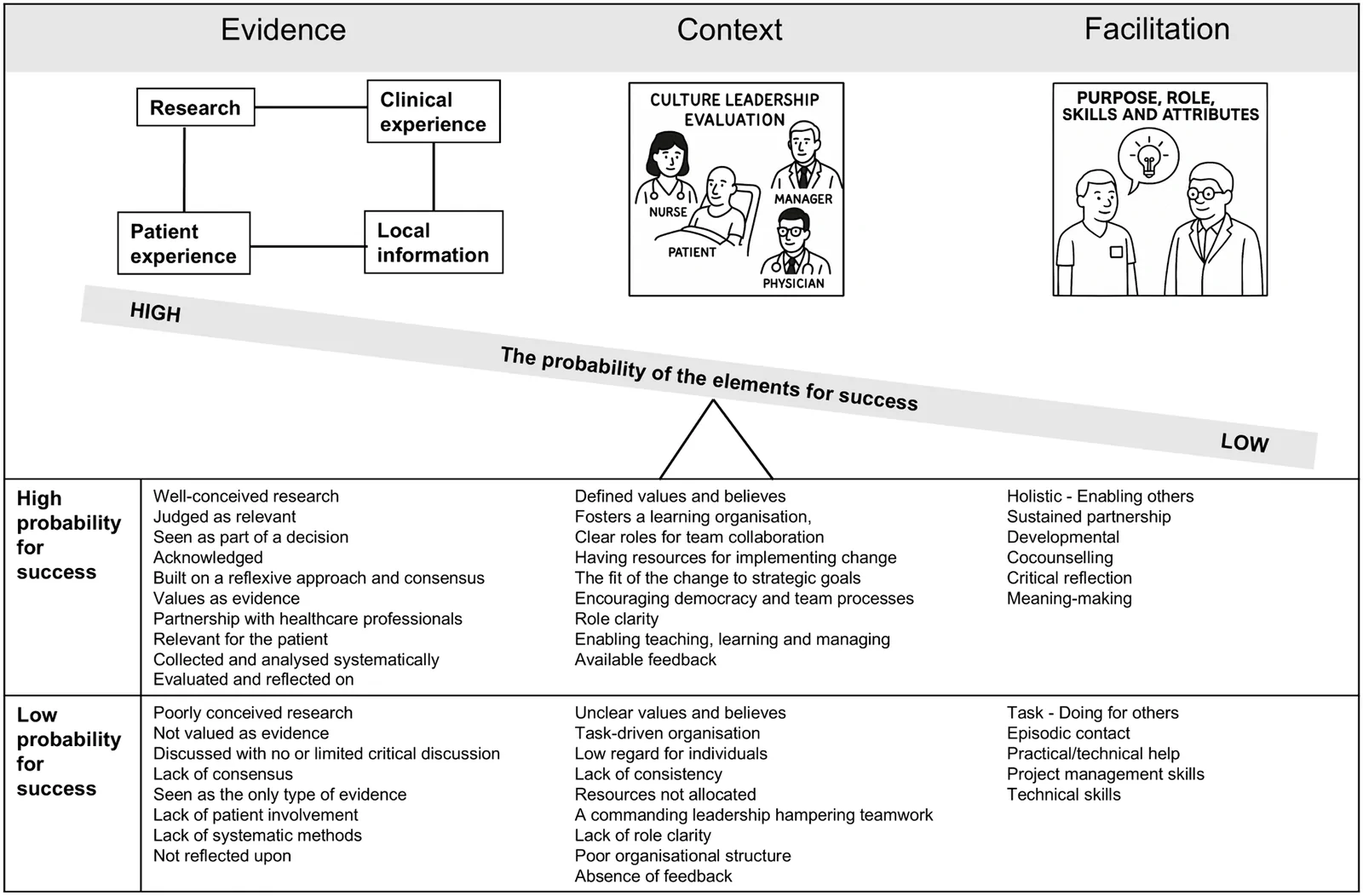
Description of the elements of PARiHS conceptual framework and factors that influence the probability for successful implementation.

The PARiHS framework [21] provided a theoretical underpinning for this study. The framework presents key aspects for successful implementation as a function of the interaction between three elements: evidence, context and facilitation. The first element *evidence* explains the support of the evidence being used, which contains four sub-elements: research evidence, clinical experiences, patient experiences and local information. The second element *context* concerns the quality of the context, which include tree sub-elements: culture, leadership and evaluation. The third element *facilitation* relates to factors that enables a positive change process, which comprise two sub-elements: roles and skills and attributes. The PARiHS elements with their associated sub-elements are each positioned in a high or low continuum, which explains the success for implementation of evidence [21] (Fig 1).

The PARiHS conceptual framework [21] was used in accordance to the deductive analysis approach [20] in order to logically organise the large amount of study data in correspondence to our study aim and research questions. The choice of organising data by use of the PARiHS conceptual framework [21] aimed to help readers understanding of factors that facilitate or hinder implementation of nurse-based interventions into clinical settings. This kind of information may prevent possible pitfalls in future nursing implementation studies.

### Study setting

**Fig 2:**
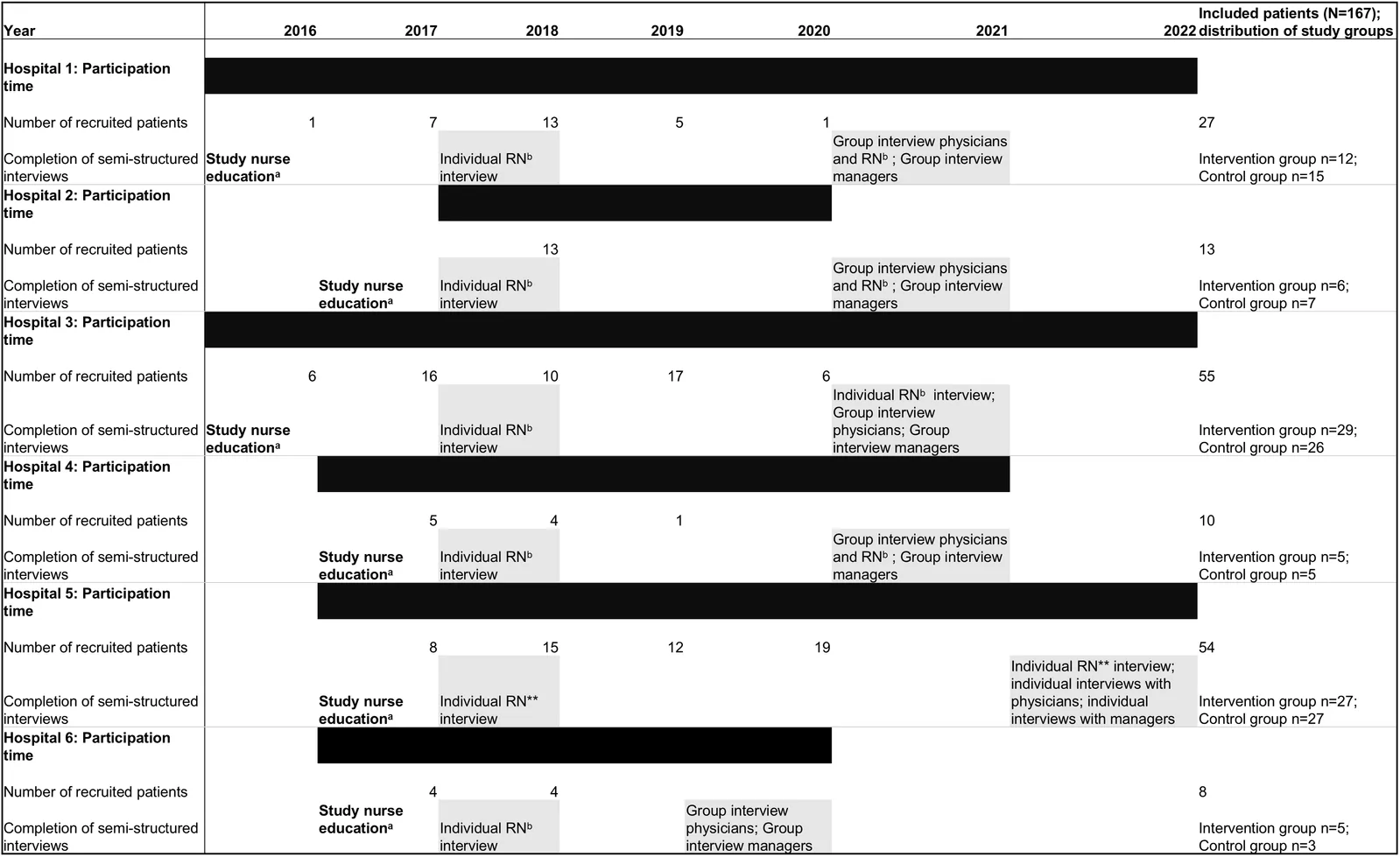
Timeline of the RN-based intervention study. Reported per hospital**,** data collection time points and study informants. ^a^ = one-day training session before the intervention, a three-day course in motivational interviewing and communication and four tutorial meetings during the study period. ^b^ RN = Registered Nurse.

Six publicly funded Swedish hospitals, two county hospitals and four university hospitals situated in mid and south Sweden, participated in the intervention study. Although the intervention was in line with their goals, the hospitals lacked a nurse-led follow-up program. Table 2 provides an overview of the roles of informants in the present study prior to the start of the intervention. The intervention study was approved by managers, physicians and the RNs before it took place from November 2016 to December 2022. The goal of the intervention study was to recruit 500 patients to participate. However, the recruitment only resulted in a cohort of 167 participating patients [15,16]. Recruitment of patients in clinical settings was challenging, which further supports the aim of the present study. The participation per study clinic varied from three to seven years (Fig 2).

**Table 2:** Study informants’ roles at the outpatient clinic before and during the intervention study.

	Managers	Physicians	Registered nurses
**Before the study:**			
***Role***	- *Manager of the hepatology unit* were responsible for the medical aspects of care and physicians. - *Ward managers* were responsible for nursing care staff.	To deliver standard of care to patients.	Administrative role: - telephone consultations on demand - planning the medically based standard of care delivered by physicians.
***Study approval***	Written agreement to make resources for the intervention study available.	Mutually agreed to the medical aspects of the changed routine according to the study protocol.	Mutually agreed to: - Implement the nurse-based follow-up routine according to the study protocol. - Undergo education to perform the QLiNCaM^a^ intervention.
**During the study:**			
***Role***	- Manager of the hepatology unit remained unaffected during the intervention study. - Ward managers received yearly feedback about the study progress.	- Identify participants who fulfilled the criteria for inclusion in the intervention study. - Provide standard medical follow-ups for all patients regardless of their study group. - Serve as a consultant for the trained RNs^b^.	*Without specific knowledge of the intervention*: - obtained informed consent - performed baseline visit for randomisation of participants. - Responsible for collecting study data from patients in the control group.
			*Trained to perform the intervention:* - Had a special interest in LC^c^ nursing. - Responsible for implementing the intervention. - Responsible for collecting study data from patients in the intervention group.

^a^the QLiNCaM=The Quality Liver Nursing Care Model.

^b^RN = Registered nurses.

^c^LC = Liver cirrhosis.

### Roles and implementation of the intervention study

The intervention study had a randomised controlled study design, which meant that patients in both the control group and the intervention group were treated at each outpatient clinic. Adjustments were made to reduce the influence of the intervention on the control group. The greatest risk of this occurring was when the trained RN responded to patients during telephone consultations on demand or cared for patients during paracentesis or endoscopy. Physicians could also provide information about the intervention to patients in the control group. Therefore, only one physician per study clinic had full insight into the content of the intervention. The roles of the study informants during the intervention study are presented in Table 2. Henceforth, when mentioning RNs in the present study, we refer to those trained to perform the intervention.

### Recruitment of study informants

In the present study, seven of the eight RNs who performed the intervention and a purposeful selection of ten physicians who provided standard medical follow-up to patients provided verbal and written consent to participate. In addition, all twelve managers responsible for the physicians and nurses at the outpatient clinic approved participation (Table 3). Informed consent was communicated by e-mail and collected by the first author. One RN and one physician declined participation for personal reasons.

**Table 3:** Characteristics of the study informants.

	Registered Nurses (N = 7)	Physicians (N = 10)	Managers (N = 12)
Gender (Female/Male)	7/0	4/6	6/6
Age			
18-39	2	2	0
40-65	5	8	12
Experience of hepatology outpatient care			
< 2 years	2	0	1
3–10 years	2	5	0
> 11 years	3	5	10
Missing	0	0	1
Employed at:			
Hospital 1	2	2	2
Hospital 2	1	1	2
Hospital 3	1	2	2
Hospital 4	1	1	2
Hospital 5	1	2	2
Hospital 6	1	2	2
Interview type:			
Individual	3	2	2
Group	0	8	10
Both individual and group	4	0	0

### Data collection

Swedish hospital care is politically controlled and regulated per region. Political decisions may have influence on the economy and healthcare resources. Due to the long period of participation for each study clinic, data was collected at two time points per hospital to cover any changed directives or clinical challenges. The first individual semi-structured interviews (n = 7) concerned the RNs’ new role and were performed with RNs (n = 7) from November 15^th^. 2018 to January 25^th^. 2019 (Fig 2). They included six questions about the RNs’ changed role in the organisation, thoughts about the intervention and its influence on standard outpatient care (S1, Table 1). Data were collected face-to-face or by telephone by a master student, trained in interview techniques, but not otherwise involved in the intervention (Fig 2).

The second semi-structured interviews concerning implementation of the intervention and performance of the study as a whole were conducted from December 17^th^. 2019 to May 20^th^. 2022. Depending on the organisational situation these were individual or group interviews (RN and physician interviews n = 8; manager interviews n = 7). Groups included RNs and physicians or two managers. The data were collected by two RNs without other involvement in the intervention. Individual interviews were performed by one interviewer, whereas group interviews were performed with one interviewer and one observer present. The observer assisted with time control, recording of the interviews and probing questions when necessary [22]. All interviews included topics covering the primary and secondary research questions and included seven (RN/physician interview guide) or five (manager interview guide) questions about the study performance and climate at the clinic, with follow-up probing questions. The questions were inspired by the Consolidated Framework for Implementation Research [23], which covers broad aspects of implementation (S1, Table 1). The interview guides were tested in pilot interviews before data collection, which resulted in reformulation of two questions.

In total, data from the 29 informants were contained in twenty-two semi-structured interviews, representing a total of 881 minutes (mean = 40 minutes). Nineteen interviews were performed via Zoom, two face-to-face and one by telephone. Twelve interviews were individual and ten were group based. The RNs were interviewed on two occasions, whereas physicians and managers were interviewed once (Fig 2). All interviews were audio recorded.

### Data analysis

The interviews were transcribed verbatim and analysed as a single dataset from the outset. Directed content analysis was performed in four steps as described by Hsieh and Shannon [20]. In step one, a coding scheme was employed in accordance with the PARiHS conceptual framework [21]. During analysis the elements: evidence, context and facilitation with their associated sub-elements of the PARiHS conceptual framework constituted a lens. In the second step, the transcribed interviews were read. All statements that corresponded to the study aim were highlighted, labelled with a code that reflected their content and then sorted into the predetermined PARiHS code scheme.

In step three, all codes were reviewed and sorted into two groups. Codes in the first group indicated a high probability of implementation success and the second group a low probability of implementation success. Steps one to three were conducted by the first author and reviewed by the second author. In step four, the authors searched for categories that reflected the data under the predetermined PARiHS elements. The authors worked iteratively to assign codes to the transcribed text and discussed the analysis repeatedly until consensus was reached. NViVO® software was employed in the analysis.

### Ethical considerations

The study followed the principles of the Helsinki Declaration [24]. Permission was obtained from the employer to interview employees. Informants gave their informed consent. The Regional Ethics Board in Uppsala, Sweden approved this study, ID 2016/146. The informants received no compensation for their participation.

### Rigour and reflexivity

The four concepts of trustworthiness: credibility, dependability, confirmability and transferability were addressed in accordance with Lincoln and Guba [25]. Credibility was ensured by the comprehensive amount of data totalling 881 interview minutes and representing RNs, physicians and managers at all study clinics. Furthermore, data covered all the PARiHS elements and 7 of its 9 sub-elements [21] that corresponded with the study aim. During the analysis we strived to ensure transparency and used an iterative process. The coding process implied highlighting statements referring to the high or low PARiHS continuum, about which the authors held repeated discussions to reach consensus and achieve dependability. Confirmability concerns the researcher’s objectivity during the study process. The first author participated in the design and implementation of the intervention and had regular contact with all study clinics during the implementation and follow-up phase. Two RNs, who were not involved the project, performed all interviews to reduce the risk of influencing informants. Although the first author conducted the first analysis, the second author played no part in the intervention and could therefore ensure neutrality during the repeated discussions. Quotations from the study informants may help readers to further judge the confirmability. Transferability of the study results has been enabled by the thorough description of the study setting. Adherence to the EQUATOR guideline Consolidated criteria for Reporting Qualitative Research guidelines (COREQ) (S2) [26] was employed to consolidate methodological rigour and thorough reporting.

## Results

### Characteristics of the sample

The informants represented RNs, physicians and managers from all six study clinics recruiting patients in the QLiNCaM intervention study. Most of the informants were female and had more than 11 years of experience from hepatology outpatient care, of which the managers were most experienced (Table 3).

### Aspects of implementing nurse-based follow-up

The results reported below follow the three key PARiHS elements, with associated sub-elements in headings and sub-headings respectively. Quotations representing the high and low PARiHS continuum are presented in Table 4.

**Table 4:** Overview of the deductive analysis process.

Statements indicating high probability	Statements indicating low probability	Category	PARiHS elements
			
Q1: When… this intervention started, … then … you realise how important nursing is… when treating LC^a^ patients (Manager 8)		Good timing	***Evidence*** Research evidence
Q2: We need to be more proactive …not wait for them to come when they are really ill. Instead catch them earlier by means of more frequent contact with the nurse. … That is for patients perceived as being at risk of deterioration. (Manager 3)		A need for RN-based^b^ LC^a^ follow-up	Clinical experience
Q3: We talk about issues besides the medical aspects, e.g., sleep problems, how the illness affects the family, relatives, psychosocial factors of concern, work, lifestyle changes. As a nurse you have more time to discuss such matters compared to the physicians. There are more opportunities to talk about smoking, alcohol and other issues that need attention. (Registered nurse 7)	Q4: We managed to include some relevant candidates for the intervention. Many possible candidates declined participation. So those who finally participated were perhaps not those most in need. (Registered nurse 4)	A desirable addition to the present healthcare service	
			***Context***
Q5: Before…. nurses were more passive than physicians. Lately I have noticed a change. The nurses are more interested in development and new processes. So, things are changing. (Manager 2)	Q6: We haven’t had a well-functioning clinic for the last 1.5 years. And in our hepatology team we discuss what is going on, the need for change and what we must improve. But nothing is happening. Our focus is on acute procedures. All elective activities are put on hold. That’s the way it is. (Registered nurse 4)	A quest to be a learning organisation	Culture
Q7: It [RN-based intervention] fits well in our strategies at the gastrointestinal outpatient unit. It is in line with our ideas for development. It fits like a glove. (Manager 3)	Q8: I found it really hard giving excellent care to the intervention group and then NOT to those in the control group. To me it was unethical. This study outlines how to perform the follow-up. And I am stuck in an ethical dilemma because I want to provide this care to everyone. Somehow, I must respect the study protocol, but I find it really difficult. I don’t know what approach to adopt. At the same time, I really want to improve care for patients in real need. (Registered nurse 2)	A feasible and appropriate design for the clinical setting	
Q9: It [RN-based^b^ intervention] raises the competence level among the nurses. They can then teach the rest of the nurses. So, increased competence is what I appreciate the most. (Manager 7)	Q11: At a superficial level it is about decompensated cirrhosis, health consequences and guidelines. I don’t know the details. More about what we follow and when. That’s all I know. (Physician 5)	Was something in it for me?	
Q10: Everyday, we notice that we make a difference for these patients. The fact that they [the patients] can turn to us. (Registered nurse 5)			
Q12: We have great communication and collaboration. If I ask a colleague to change tasks it happens. In that way, they are amazing. Even if not involved at all in the project. (Registered nurse 2)	Q13: Teamwork depends on different personalities. As in most workplaces, sometimes it works fantastically and sometimes less well. Communication doesn’t work. The reason is not our organization but the different personalities in the team. (Manager 3) Sometimes it works poorly due to the chemistry in the team. Among both the physicians and the nurses. (Manager 4).	Support by means of team collaboration	
	Q14: Nobody notices our performance. We are great at following up the liver patients, but we never get a medal for that. (Physician 1) Just as in previous years it is the IBD^c^ treatments and the colonoscopies that count. (Physician 2)	Fragility due to structural barriers	
Q15: Now we know we have the skills. When the study is over, we will continue the nurse-led follow up. We can simply go on. This study has really helped us. We don’t need to reinvent the wheel. (Manager 8).	Q16: Some patients don’t view themselves as seriously ill. Some of them live far away from the hospital. We have a large catchment area. If they have other caregivers, they end up with many hospital visits. Sometimes we send the information letter too soon, immediately after they are diagnosed and admitted to hospital. Perhaps they haven’t had time to grasp the new situation. (Registered nurse 3)	Confirmed the value of the intervention	Evaluation
Q17: When a new study is presented, my basic approach is that it should be done. I wish to facilitate research at my unit. However, I also need to review the costs, the financial impact or any ethical considerations. I need to form my own opinion. What is the scientific benefit? Even if I am not familiar with the area of interest, I want clarification about the benefits. Only the manager is responsible for resource utilization. (Manager 4)	Q18: Making space for all kinds of research is problematic. Finding time is a challenge. Even if it is a good cause and very important, time is taken away from other patients. (Manager 11)	A risk-and-benefit assessment was fundamental for clinical approval	Leadership
	Q19: This project started without the complete involvement of the physicians. When these patients visited the ward or the consultant there was a lack of understanding about the hepatology nurse’s new tasks and how to optimize her delivery. I guess it was my responsibility. It never worked even when I informed the physicians about the protocol. Thus, few patients were seen by the nurse. (Manager 2)	Managers did not make sufficient efforts	
			***Facilitation***
Q20: I really appreciate this network for those of us who are involved in this study. It adds a lot and is a great support. So, I find the support I need here, and it is perfectly adequate. (Registered nurse 2)		Individual knowledge and common consensus was gained by external facilitation	Role
Q21: We [registered nurse and manager] decide in a dialogue how to determine the best approach, the number of days required for the intervention based on the need. It varies a lot. (Manager 9)	Q22: My manager was informed and approved that we would implement the nurse-led follow-up. But then it was disregarded. I was supposed to spend half a day per week performing the intervention. But during the last few years no time was allocated. (Registered nurse 5)	Managers’ role for internal and structural facilitation	
Q23: The project gave extra motivation. We have planned for what we want to achieve and why (*high* PARiHS continuum). When other big things have been going on it has been difficult to get the mandate (*low* PARiHS continuum). However, the project enabled us to work on things where we already had a structure (*high* PARiHS continuum) (Physician 5)		A need to be assigned a mandate	Skills and attributes
Q24: My professional role is expanded, more autonomy and more confidence. It’s always interesting to learn new things and participate in a study. Your performance improves. You manage previously difficult things. Professional growth is a positive experience that makes you strong. You get the courage to decide independently without consulting the physician all the time. You trust your own competence to give the best possible follow-up for the patients. (Registered nurse 4)	Q25: It’s challenging to be an autonomous nurse. And you need to challenge yourself to develop. It’s also a challenge to make sense out of these new routines and align them with current practice. Sometimes I feel stressed because I can’t fully cope with the new routine. (Registered nurse 4)	A professional growth process	

^a^LC = liver cirrhosis.

^b^RN-based = registered nurse based.

^c^IBD = inflammatory bowel disease.

#### The PARiHS evidence element and associated sub-elements.

Data reflected two of the PARiHS evidence elements [21]: *research* and *clinical experience*, but not *patient experience* and *local information*.

The sub-element *research evidence* was rarely mirrored in the data. However, good timing of the implementation of RN-based follow-up for LC patients and positive reports from similar initiatives in Denmark were reflected in the data, adhering to the *high* PARiHS continuum (Q1, Table 4).

The sub-element *clinical experience* reflected how the informants perceived a need for RN-based LC follow-up. The patients were considered frail, stigmatised and severely ill and therefore expected to benefit from proactive outpatient care (Q2, Table 4). The informants were concerned about patients’ limited knowledge of their disease and individual needs for repeated information. The informants expressed that the intervention constituted a desirable addition to the present healthcare service, which contributed to enhanced quality of care, exemplified by improved access to outpatient care, which facilitated rapid assessment of patients and identification of early signs of complications. The RNs experienced a new holistic way of communicating with patients, which left room for the patient’s agenda (Q3, Table 4). The patients’ needs were identified via the person-centred approach and the recommendations adapted accordingly. The intervention also applied a new structure to their encounters with patients, for example by the use of tests for hepatic encephalopathy, which could be applicable to other patients at the outpatient clinic. RN involvement contributed to stability in the team and improved caregiver continuity for the patients. The major findings pertaining to the informants’ clinical experiences of the intervention represented the *high* PARiHS continuum. There were few statements representing the *low* PARiHS continuum. These reflected the limitation that RN-based follow-up was only delivered as part of the randomised controlled study, which restricted the intervention to half of the patients who had agreed to participate. Patients who needed the intervention most tended not to participate (Q4, Table 4). The structured form of the intervention was more time consuming than care as usual. Some RNs experienced that the initial patient meetings were overly regulated by the protocol, which limited individualization in line with the patient’s needs.

#### The PARiHS context element and associated sub-elements.

All sub-elements of the second PARiHS element *context* [21], including *culture*, *evaluation and leadership*, were represented in the informants’ narratives.

The sub-element *culture* was described as coloured by a quest to be a learning organisation and to achieve evidence-based practice, which was especially emphasised by managers and physicians. The climate was sometimes described as enthusiastic about keeping track of new guidelines and discussion forums, which resulted in decisions about how to act on the new medical guidelines. After primary discussions by physicians and managers, RNs had shown increased interest in becoming involved in these team discussions (Q5, Table 4), all reflecting the *high* PARiHS continuum. The *low* PARiHS continuum was represented by contrasting comments that implementation was sometimes based on individual interests or that implementation had been deprioritised during the study period due to the high workload (Q6, Table 4).

The informants confirmed that the intervention design was appropriate and feasible for the clinical setting. They acknowledged that the intervention was in accordance with or helped them to achieve their strategic goal of involving RNs in LC outpatient care (Q7, Table 4). The procedures to implement the intervention were easy and could be performed independently by the RNs. All these experiences represented the *high* PARiHS continuum. In contrast, the *low* PARiHS continuum concerned recruitment of participants. Four of the six hospitals found it difficult to establish routines for patient recruitment. Therefore, the recruitment was slower than expected, which ultimately prolonged the duration of the study. The fact that patients in both the intervention group and the control group were cared for in the same clinical setting sometimes implied logistic problems with separating the groups. The RNs mentioned that their role isolated them from other colleagues. Therefore, they expressed a wish to share the responsibility with a colleague. Occasionally, the RNs had to face disappointed patients, who were allocated to the control group during the randomisation process. The RNs could also be afraid of burdening patients in the intervention group with too many visits or challenging tests during the data collection. Wanting to improve the evidence for RN-based outpatient care, while at the same time being unable to help all patients was described by the RNs as an ethical dilemma (Q8, Table 4). RNs at some hospitals did not have a permanent office and had to use an empty room for the day, which could be stressful.

Another part of the culture concerned informants’ statements that they valued the intervention by asking themselves the question ‘what’s in it for me?’, which led to personal and/or professional satisfaction. The managers valued the RNs’ noticeably higher motivation for work and acknowledged their enhanced competence (Q9, Table 4). The physicians’ attitude towards the intervention changed during the study, from initial reluctance to fully accepting the intervention and the RNs’ new role. The physicians experienced being disburdened of their workload when responsibility was transferred to the RNs. They also appreciated the fact that the RNs could complement the medical care with longer patient conversations. The RNs reported feelings of making a difference to the benefit of patients in the intervention group (Q10, Table 4), which also was acknowledged by managers and physicians. The RNs stated that the new knowledge from the intervention had strengthened their professional role, which had been an eye-opener for the other team members about of the strengths of nursing. RNs became recognized for playing a key role in the LC outpatient team. All these aspects can be found on the *high* PARiHS continuum. In contrast, physicians’ statements about having little knowledge of the RNs’ role belongs on the low PARiHS continuum (Q11, Table 4).

Support by means of team collaboration was another aspect mentioned concerning the context. Before the study started, discussions were held to define the roles and responsibility of the two study groups within the RN team. Thereafter, managers and RN colleagues had an important role in allocating time for the study during working hours (Q12 Table 4). Over time, the RNs established a trusting relationship with both patients and physicians, which facilitated team discussions. The shared responsibility and collegial discussions were perceived as supportive by the RNs. Although the physicians had a limited role in the intervention, most of them showed commitment by identifying patients for the study and were often available for RNs’ questions during or after patient visits. These aspects of team collaboration and support are on the *high* PARiHS continuum. On the other hand, the *low* PARiHS continuum was associated with some RNs’ experiences of lack of support from physicians. Although they sought cooperation, they initially sensed resistance from some physicians, or that the physician was insecure or reluctant to let RNs take responsibility (Q13, Table 4).

The informants mentioned fragility due to structural barriers when implementing the study in the clinical setting, which represented the *low* PARiHS continuum. All hospitals struggled with a shortage of RNs, which reduced the opportunity for RNs to participate in clinical research. Re-organisations at some study clinics led to an even more extensive workload, which forced the managers and staff to prioritise. Sometimes there was a conflict of priorities between different gastrointestinal patient populations and the informants reported that traditionally, patients with liver disease had the lowest priority (Q14, Table 4). The high workload meant the RNs worked with the study when time was allocated, which consumed their energy. During the Covid-19 pandemic, the time devoted to the study was minimal, the recruitment rate was zero and the intervention mostly conducted by telephone for needy patients. Another structural barrier was a shortage of involved and responsible RNs in the study. During the study period, some RNs retired, which meant a loss of competence and a time-consuming process to search for and teach a new colleague about the RN’s role in the study.

In the sub-element *evaluation* informants’ narratives confirmed the value of the intervention for both the outpatient clinic and the patients. The RN-based intervention was experienced to have developed outpatient care in a positive direction and improved the utilisation of existing skills and resources. By participating in the study, managers, RNs and physicians had learnt important aspects of the role of RN in outpatient LC care, which could now be continued (Q15, Table 4). The new knowledge was also considered important for future developments that could be applied to other patient populations. The value for the patients was exemplified by patients showing interest and expressing satisfaction about the regular meetings with RNs, which increased their involvement. These statements represented the *high* PARiHS continuum. The *low* PARiHS continuum included a concern about patients who declined participation and therefore did not receive RN outpatient care during the study, as the informants thought that they could benefit from the intervention. Reasons for declining participation were feeling healthy, having no concerns about their liver disease, living far away from the hospital or feeling too ill and therefore visiting the hospital frequently (Q16, Table 4).

The sub-element of *leadership* was contained in the managers’ descriptions of a risk-and-benefit assessment that was fundamental for the clinic’s approval of the intervention study. Statements that applied to the *high* PARiHS continuum were the managers engaging in the intervention by gathering information and reading the study protocol in advance. They evaluated the burden of the intervention in terms of RN working hours, financial aspects, resources and ethical issues (Q17, Table 4). They accepted the intervention study based on its low burden on the overall organisation and that the patients needed care at the outpatient clinic regardless of the study. In contrast, the *low* PARiHS continuum was represented by the managers’ limited knowledge of previous evidence of RN-based interventions for patients with LC. In addition, they prioritized financial outcomes and reduced burden on physicians, rather than patient-related outcomes and RNs’ work situation. Although the study was accepted in advance, the managers stated that financial restraints had been problematic during the study period (Q18, Table 4). The managers were concerned about a competitive situation that occurred during the study, which involved the increased interest in RN-based outpatient care for LC patients from other hospitals. This simultaneous process prevented the study from continuing. Some managers did not make sufficient efforts, and a majority of managers expressed uncertainty about how much knowledge they needed about the study and were concerned about influencing the results by their actions. When necessary, the managers made themselves available to support the RNs in problem-solving but delegated the responsibility for implementing the study to the RNs. The managers reflected on the physicians’ limited knowledge about the intervention study and their failure to ensure that physicians were informed about the study in advance, which had influenced the recruitment of participants and made the new RN role unclear (Q19, Table 4).

#### The PARiHS element of facilitation and associated sub-elements.

Both sub-elements of the third PARiHS element *facilitation* [21], including *role* and *skills and attributes*, were represented in the informants’ narratives.

The sub-element *role* included RNs**’** narratives of how their individual knowledge was enhanced by training and how consensus was achieved by external facilitation. The tutorial sessions provided by the first author were stated to be of high importance. These meetings had deepened their knowledge and led to consensus about the RNs’ role in LC outpatient care (Q20, Table 4), all representing the *high* PARiHS continuum.

The first line managers had a role in the internal and structural facilitation of the intervention study at the outpatient clinic. During the establishment phase, the first line managers participated in start-up meetings and enabled RNs’ participation in the regular tutorial sessions. During the study period, some of the first line managers remained positive, held continuous encouraging dialogues with the RNs and reminded colleagues about the study in various forums. Furthermore, they customized the RNs’ schedules to create time for patient meetings (Q21, Table 4). These statements represented the *high* PARiHS continuum. Although the managers strongly wished to be supportive, it was sometimes difficult for them, which were examples of the *low* PARiHS continuum. The difficulties resulted from the changed conditions over the six years of the study, which at some hospitals resulted in a lack of space and resources for carrying out the study and participating in tutorial sessions (Q22, Table 4). The managers occasionally forgot to remind other staff members about the study. According to the participating RNs, if the new RN role is to be successful, it is essential that managers clearly prioritise it.

The sub-element *skills and attributes* was reflected in the establishment phase of the study. In the early phase of the study the RNs needed to be assigned a mandate to implement the intervention at the outpatient clinic, which was provided by the first line managers. Participation in the intervention study enabled one clinic to argue in favour of a new RN-based routine, which helped to provide some structure in situations where the incentive to continue the RN-based outpatient care was questioned. Mandating the RNs was associated with the *high* PARiHS continuum, whereas the sudden change in conditions for conducting the intervention and striving for the implementation of RN-based outpatient care could rapidly ruin the RNs’ mandate and was one example of the *low* PARiHS continuum (Q23, Table 4).

The RNs explained how they grew into their new role in a stepwise manner, viewed as a professional growth process. The new way of working and being part of a clinical study meant the RNs needed time for documentation and reflections, which was an important factor that had to be accepted by colleagues and managers. The RNs**’** involvement in the intervention increased their responsibility, which required education. The education they received before the study started and continuously during the study strengthened their own competence and ability to provide independent counselling for patients. Their growing competence and being part of a large clinical trial also implied a sense of professional pride and a stimulating work situation. During the study period, the RNs expressed that the responsibility of being the interventionist had increased their confidence in the delivery of independent LC nursing care (Q24, Table 4). These positive experiences are examples of the *high* PARiHS continuum. In contrast, the *low* PARiHS continuum was reflected in the RNs’ special competence, which meant that there were few other colleagues who could support or collaborate with them during the intervention. At the beginning of the study, the RNs sometimes experienced the individual patient meetings as challenging and stressful. These feelings were exemplified by situations when they felt uncertain about what a patient meeting should include or when they were concerned that they might misjudge signs or symptoms (Q25, Table 4).

## Discussion

**Fig 3:**
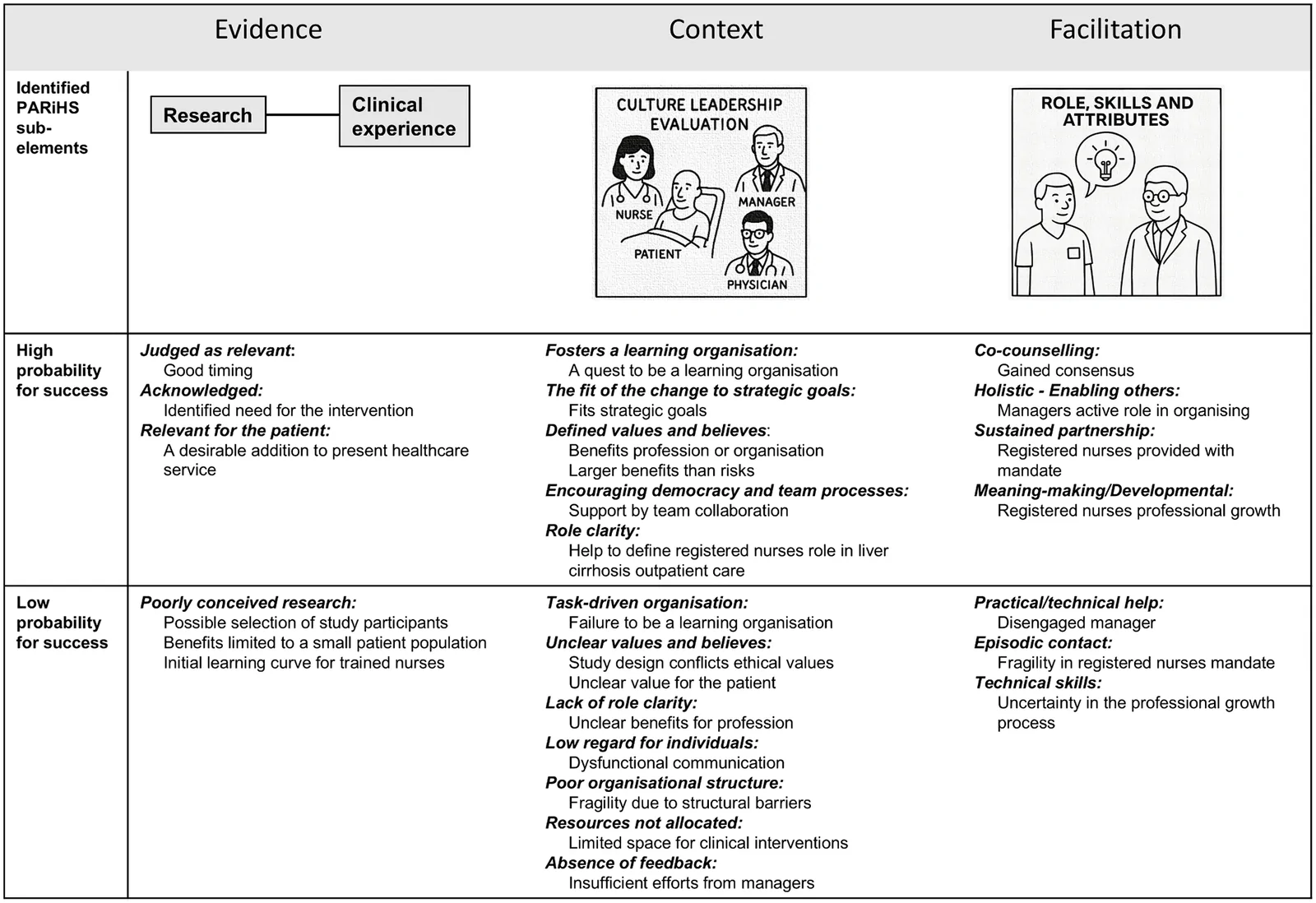
Overview of the study results in relation to the PARiHS elements. The definition of the identified high and low attributes for each PARiHS element is written in bold and italic, with related study finding in groups below.

The results presents important aspects of the implementation of RN-based outpatient care in the Swedish healthcare context. All PARiHS elements, and seven of the sub-elements [21], were represented in the data. We found several factors of high and low probability for success of the QLiNCaM implementation. The predefined elements in the PARiHS conceptual framework in relation to the study findings is visualised in Fig 3.

The most important findings concerned how the implementation influenced RNs’ professional development and ambition to improve outpatient care. The RNs received new tools, a nursing model and mandate to implement RN-based care for patients with LC, which increased their professional motivation. Similar to the implementation of a nursing model in inpatient surgical wards [17], implementation of new nursing models implies a learning process and familiarisation with the new routines, which needs support from managers. In addition to these findings, the present study reveals the importance of receiving support from physicians. The intervention increased RNs’ competence and autonomy, which previously has been highlighted as an important factor for an attractive RN workplace [27]. However, the RNs’ mandate at some hospitals in this study turned out to be fragile. Changed conditions at the outpatient clinics implied that the RNs were instructed to focus on the core mission of the outpatient clinic. Even though the intervention was well aligned with the common goals of the outpatient clinics, the benefits of the intervention were disregarded. In accordance to Jabonete and Roxas [28], the experiences from the present study has been described as frequently occurring structural barriers in three-fourth of nursing intervention studies overall in clinical nursing research. Therefore, the present result highlights the fact that the mandate for and autonomy of RN practice is influenced by the organisation, leadership and the medical profession.

There are national incentives to provide close and accessible healthcare for Swedish citizens, which include person-centred and team-based care [12,29]. Based on the present findings, we are concerned that when it comes to savings and reorganisation, medical aspects of care are prioritised over person-centred and team-based care, which could potentially overturn national decisions. Hence, there are reasons for questioning whether Swedish healthcare is receptive to a higher level of ambition among RNs? RNs involved in this intervention study successively grew into their new role with increased authority and accountability. The negative situation at a few of the involved hospitals meant that some RNs left their employment, meaning that the study terminated earlier than planned. In contrast to Ahlstedt et al. [27], findings of aspects that keep RNs in the profession, such as their autonomy and competence were limited, which in line with Bahlman-van Oojen et al. [30] is a reason for RNs leaving their employment. In today’s financially strained Swedish healthcare system, the light at the end of the tunnel is that the RNs and managers expressed awareness of research ethics. However, the present study revealed that the utility-evaluation of the intervention on both an individual and professional basis was deemed superior to the scientific value, which in concordance to Jabonete and Roxas [28] may endanger clinical nursing research.

Most of the informants had experienced challenges with implementation in general and during implementation of the intervention study. In concordance to Roberts et al. [18] and the use of the PARiHS conceptual framework [21] in this study revealed information about important factors that need to be taken into consideration when implementing a clinical RN intervention. Evidence from outpatient nursing in other chronic illnesses was not mentioned, which can be interpreted as a culture that is inherently ignorant regarding evidence for outpatient nursing care. The clinical experiences showed the positive quality of care aspects, which has been confirmed in previous studies [16]. Nevertheless, the study was difficult to perform due to the risk of a sudden lost mandate when the organisational climate changed, which is a previously identified risk for nursing research globally [28].

The evidence-based practice culture seemed to vary considerably between the outpatient clinics. Some hospitals had structured discussions about evidence and a decision-making forum, whereas at others, evidence-based practice was dependant on individual interest. RNs rarely attended these almost exclusively medical discussions. In general, nursing research was neither asked for nor encouraged by the managers. Providing evidence-based practice is a requirement stated in the first paragraph of the Swedish Patient Act [31]. We interpret the present results as an indication of low priority and limited resources allocated to clinical nursing research by Swedish stakeholders. This is a major concern that makes the Patient Act a taken for granted cliché.

### Strengths and limitations

One limitation of a qualitative study design is the impossibility of making comparisons. We refrained from comparing the experiences of RNs, physicians and managers, which was based on a decision to describe the conditions for implementing a nursing intervention in a clinical setting, in which all these professions were represented. There were differences in data collection procedures. Different questions were asked in the interviews, which comprised both individual and group interviews. We chose to see these circumstances as a strength, as interview questions need to be adapted over time and relevant to the informant. In addition, we were flexible when arranging the interviews by taking account of the informants’ situations. The large amount of data should be seen as a strength. Due to the study period of six years for the intervention study, we consider repeated interviews to be a strength that provided information about how conditions may change within the healthcare organisations over time.

The results of this study reflect the evidence, context and facilitation of RN-based LC outpatient care in Sweden, which is highly relevant due to current national guidelines for LC care [32]. The results may be transferred to Swedish LC outpatient care and possibly other nursing research projects within the same culture. The deductive approach and use of the PARiHS conceptual framework as a lens during the analysis facilitated the organisation of the large amount of interview data. Furthermore, to grasp and highlight important aspects of implementation of a clinical nursing intervention, as presented in Fig 3.

### Implications for policy and practice

The *evidence,* which underpins a clinical nursing study needs good timing and a desire for change in the organisation. However, the controlled design of a randomised trial may challenge RNs’ ethical platform of providing equal care to all patients. A clinical *context* coloured by collaborative learning and goals for implementing the intervention in focus of change is vital. Furthermore, that the benefit of the change, for the individuals and organisation, exceeds the risks. The fact that an intervention reduces the physicians’ workload, may be a strong incentive in addition to patient satisfaction and quality of care. To provide RNs with a mandate to realise the intervention in practice, as exemplified in the present study by outpatient care for patients with LC, will *facilitate* implementation*.* However, the mandate may become fragile over time. Co-counselling networks, engaged managers and RNs professional growth may increase the probability of implementation success. This study contributes with awareness of possible barriers when introducing qualified RNs in the routine follow-up of patients with liver cirrhosis, which have potential to improve patient-related outcomes following nursing care [15,16].

## Conclusions

In conclusion, implementation of nurse-based interventions is facilitated by simple procedures that can be performed independently by RNs but also by the fact that the interventions are considered valuable for the clinic concerned. Staff shortage is a barrier to clinical research, and a moral dilemma might be experienced when not all patients in need of extensive care can be randomized to the intervention. An adequate number of benefits are needed to drive the success of an implementation. The PARiHS conceptual framework includes relevant perspectives required to implement evidence-based nursing practice.

## Supporting information

S1 Table Description of the semi-structured interview questions. (DOCX)

S2 Table COREQ (COnsolidated criteria for REporting Qualitative research) Checklist. (PDF)

## Abbreviations

LC: liver cirrhosis
QLiNCaM: the quality liver nursing care model
RN: registered nurse.
