# Supplementary material for: Aspects of implementing a nurse-based follow-up of patients with liver cirrhosis: A qualitative study
Source: PLoS One. 2026 Aug 21;21(8):e0356782. doi: 10.1371/journal.pone.0356782 (PMC13496453; doi:10.1371/journal.pone.0356782)
Supplement: Supplementary file 2 [file pone.0356782.s001.docx]

Supporting information 1

| **Table 1. Description of the semi-structured interview questions** | | | |
| --- | --- | --- | --- |
|  | **Broad question** | **Probing/deepening question** | **Focus area of questions** |
| **Individual interviews with registered nurses** | Tell me about yourself and your work as a registered nurse. | - Tell about your experience caring for people with liver disease. - Tell about your role in the intervention study? | Strength and weaknesses |
|  | Tell me something about the organisational conditions for the study at your clinic? | - How do you experience the prioritisation of the nurse-led intervention at your clinic? - How do you perceive the time required to complete the study? - Tell us about the personnel resources required to carry out the study? - How do you experience the support for implementing the intervention - What do you think about the venue's application for this form of patient meetings? | Goals  Streght and weaknesses  Obstacles |
|  | What is the attitude towards clinical studies in general at your clinic? | - What is your attitude towards the intervention study? - How would you describe the collaboration around this study at your clinic? - Who is involved? - How would you describe the experience of the study at your clinic so far? - To what extent do these experiences influence the implementation of the study? - Tell about occasions when you felt discouraged as a result of or in connection with the study. - To what extent do these experiences influence you in conducting the study? - How do you see the business incorporating this form of care? | Goals  Streght and weaknesses  Obstacles |
|  | Describe your role at the clinic today compared to before. | - How has the study influenced your professional role? - What has the study done for your personal development? - Do you feel you have the right skills for the task? - How do you detect when the patient needs to perform self-care? - How do you work with patients' self-care for liver cirrhosis? - How do you ensure that the patient follows your advice? | Goals  Streght and weaknesses  Obstacles |
|  | What difference do you think the study makes for:  1. the patient  2. nurses  3. doctors | When the study is eventually completed - what will the care for patients with liver cirrhosis look like at your clinic? | Goals |
|  | What advantages and disadvantages do you see with nurse-led care for patients with liver cirrhosis? |  | Goals  Streght and weaknesses |
| **Group or individual interviews with registered nurses and physicians** | Tell me how you generally approach guidelines at your clinic | - How are they presented? - How are they implemented? - How are they received? | Implementation climate |
|  | How well informed were you about the content of the nurse-led intervention? | - Where did you get the information about the intervention? - How did you stay updated? - What did you know about the content of the nurse's work with the patients in the intervention group? | Characteristics of individuals  Knowledge and belief in the intervention |
|  | Tell me how the intervention was applicable for your clinic. | - How were your resources adapted to the implementation of the intervention? - How did the intervention fit/collide with other routines? - Describe the conditions in your care environment for implementing the intervention - What other important priorities have emerged during the intervention period at your clinic? How have they affected the intervention? | Intervention adaptability |
|  | How has your manager made it possible to implement the nurse-led intervention? | - Were problems discovered during the implementation of the intervention? - How did they affect/were they resolved? - What opportunities were there to implement the intervention? How were they utilized? | Implementation climate  Leadership |
|  | How do you experience the team collaboration around patients who received the nurse-led intervention? | - Who collaborated in your team? - How did the design of the intervention affect your teamwork? | Team collaboration, communication |
|  | How do you view the patient’s knowledge about their liver cirrhosis disease? | - In comparison to others, do you see similarities? - Differences? - Describe how you engage the patients? - How do you adjust according to the patient’s preferences? - Are there similarities/differences in the way of working if you are a nurse or physician? | Characteristics of individuals  Knowledge and belief in the intervention |
|  | How do you see the role of nurses in the follow-up of patients with liver cirrhosis in the future? | - What difference does it make to - The patient? - The nurse? - The doctor? - The clinic? - What adjustments to the content of the intervention need to be made when the study ends? | Intervention adaptability |
| **Group or individual interviews with managers** | Tell me how you generally approach guidelines at your clinic | - How are they presented? - How are they implemented? - How are they received? | Implementation climate |
|  | Tell about your opportunities to support the implementation of the nurse-led intervention at your clinic? | - Tell us about events that facilitated or complicated the implementation at your clinic   - How have the financial frameworks affected the implementation? | Implementation climate  External policies and incentives,  Peer pressure, Patient needs and resources |
|  | How well informed were you about the content of the intervention? | - What is important to know as a manager? - How did you stay up to date? - How did you get the information? - Where did you get the information from? | Awareness and familiarity with the recommendation  Characteristics of the individuals  Knowledge and beliefs about the intervention |
|  | Tell me how the intervention was applicable for your clinic. | - How were your resources adapted to the implementation of the intervention? - How did the intervention fit/collide with other routines? - Describe the conditions in your care environment for implementing the intervention - What other important priorities have emerged during the intervention period at your clinic? How have they affected the intervention? | Intervention adaptability |
|  | How do you see the role of nurses in the follow-up of patients with liver cirrhosis in the future? | - What difference does it make to - The patient? - The nurse? - The doctor? - The clinic? - What adjustments to the content of the intervention need to be made when the study ends? | Intervention adaptability |
